# Supplementary material for: Platelet-derived microparticles enhance megakaryocyte differentiation and platelet generation via miR-1915-3p
Source: Nat Commun. 2020 Oct 2;11:4964. doi: 10.1038/s41467-020-18802-0 (PMC7532443; doi:10.1038/s41467-020-18802-0)
Supplement: Supplementary file 1 — Supplementary Information [file 41467_2020_18802_MOESM1_ESM.pdf]

Supplementary Information:

Platelet-derived microparticles enhance megakaryocyte differentiation and platelet generation via miR-1915-3p

Qu et al

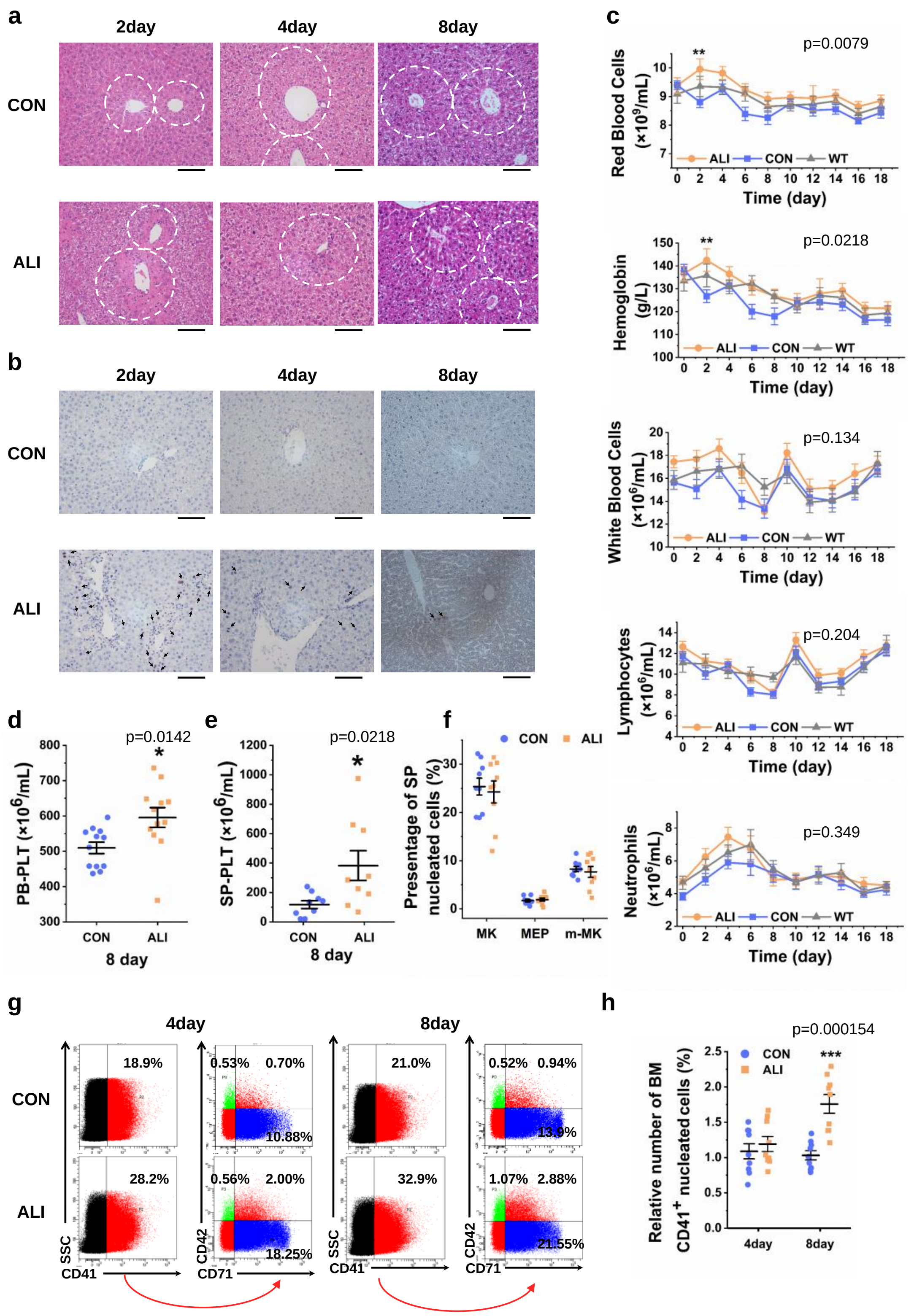

Supplementary Figure 1 CCl_4 mediated acute liver injury without hemoconcentration.

(a) Representative liver sections of control and acute liver injury mouse stained with hematoxylin-eosin. Destroyed structure was cropped out with white dashes. on day 2, 4 and 8 after CCl_4 exposure**.** Scale bar: 100 nm (b) Sections of liver collected after CCl_4 exposure were immuno-stained for the presence of Ki67 to index hepatic regeneration. Representative photomicrographs indicate positive staining in nuclei near necrotic areas (black arrows). Scale bar: 100 nm. (c) Count of red blood cells, white blood

cells, neutrophils and monocytes and level of hemoglobin in peripheral blood of the control and acute liver injured mice (n=12, two-way repeated measures ANOVA with Bonferroni's multiple comparison tests). (d-e) Platelet count in peripheral blood (n=12) (d) and spleen (e) on day 8 after CCl_4 exposure. (f) Percentage of MKs, m-MKs and MEPs of nucleated cells in spleen was measured 8 days after CCl_4 exposure. (g) A representative experiment of different differentiation stages of megakaryocytic cells from nucleated cells in bone marrow analyzed by flow cytometry on day 4 and day 8 after acute liver injury. (h) Relative number of megakaryocytes in bone marrow of ALI and control groups. Each time point in each animal cohort represents a mean ± S.E.M of 9 mice. Two-tailed unpaired t-tests were used at each time point, unless otherwise specified. Source data are provided as a Source Data file *P<0.05, ** P<0.01, ***P<0.001.

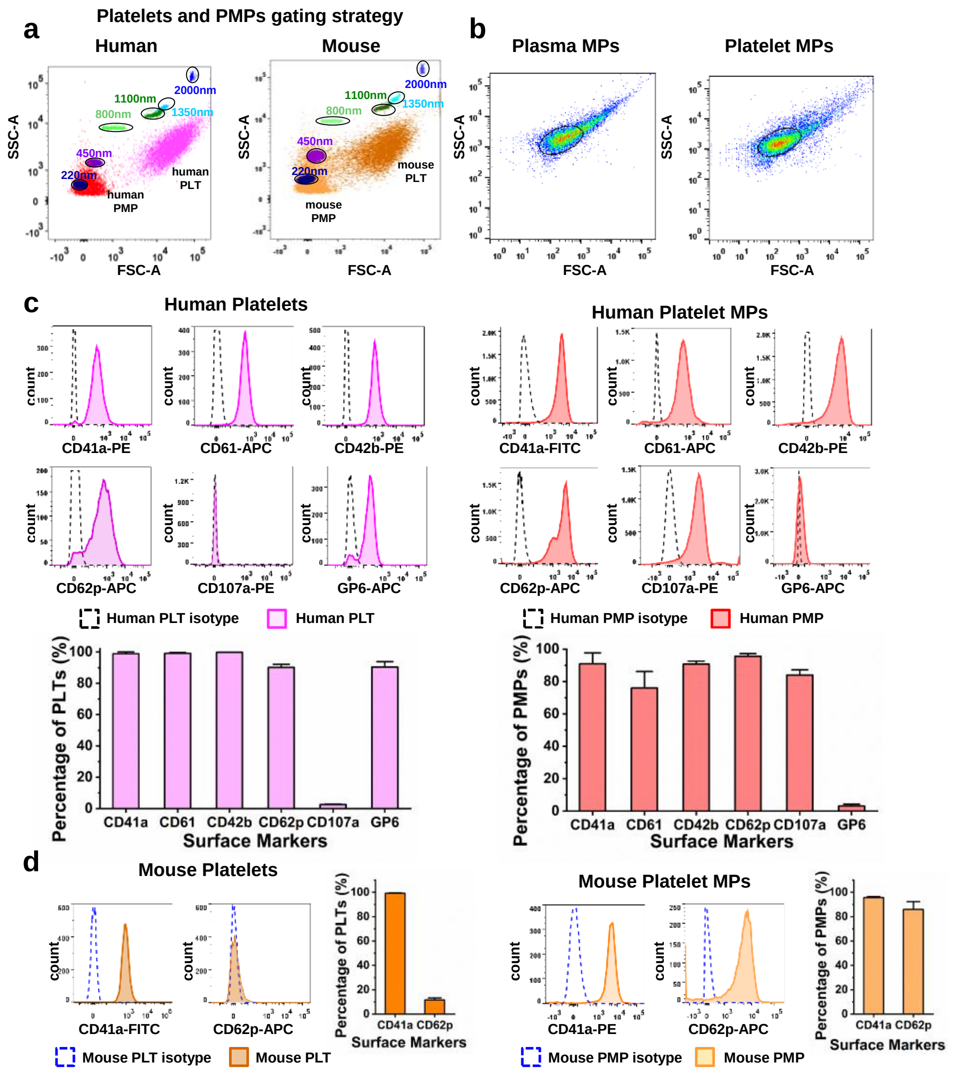

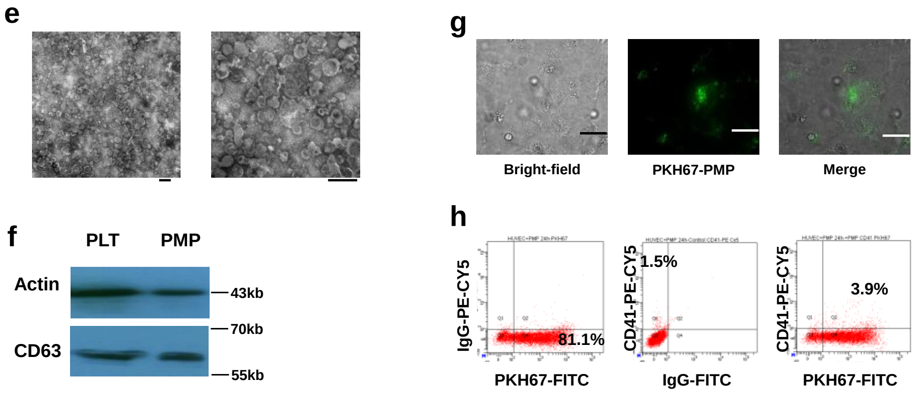

Supplementary Figure 2 Isolation and identification of human and mouse PMPs.

(a) Human and mouse isolated platelets and MPs derived from platelets were analyzed by flow cytometry. (b) Gate of human-isolated MPs derived from plasma and platelets. (c) Flow cytometric analysis showed the surface markers of human-isolated platelets (left) and PMPs (right). Since PMPs are released from activated platelets and platelets originate from MKs, PMPs are positive for megakaryocytic lineage markers and activated platelet marker. Human isolated PMPs were positive for megakaryocytic lineage markers (CD61, CD62e and CD42b) and activated platelets markers (CD62p and CD107a) and negative for glycoprotein VI. Representative images of flow cytometry analysis of isolated human PMPs are shown on the top.(n=3 donors) (d) Flow cytometric analysis showed the percentage of CD41a and CD62p of mouse-isolated platelet and PMPs. Representative images of flow cytometry analysis of isolated mouse platelets and PMPs are shown on the left. The ratio of CD41a and CD62p double-positive human or mouse PMPs was ~80%.(n=3 mice) (e) Transmission electron microscopy images showed that isolated PMPs were heterogeneous and spherical, with diameters ranging from 100 to 1000 nm (Scale bar: 100 nm). (f) Western blot examination of isolated PMPs revealed the presence of tetraspan protein CD63, a marker protein of exosomes and platelets but not of microparticles from other cells, which consisted with the report that PMPs express antigens specific to their parental cells. (g) An epifluorescence microscopy image shows that 24 hours after PMP treatment, human umbilical vein endothelial cells (HUVECs) internalized fluorescent PMPs stained with PKH-67 and exhibited diffuse fluorescence. (Scale bar: 100 nm) (h) Representative images of flow cytometry analysis of the HUVECs shows that more than 80% of HUVECs had internalized the PMPs, but less than 5% of them expressed CD41 afterwards, excluding the possibility that PMPs attached to the HUVEC surface and made the cells positive for PMP markers. All data are expressed as the mean ± S.D. Data represent three or four independent experiments performed using platelets and MPs from three independent blood donors. Source data are provided as a Source Data file

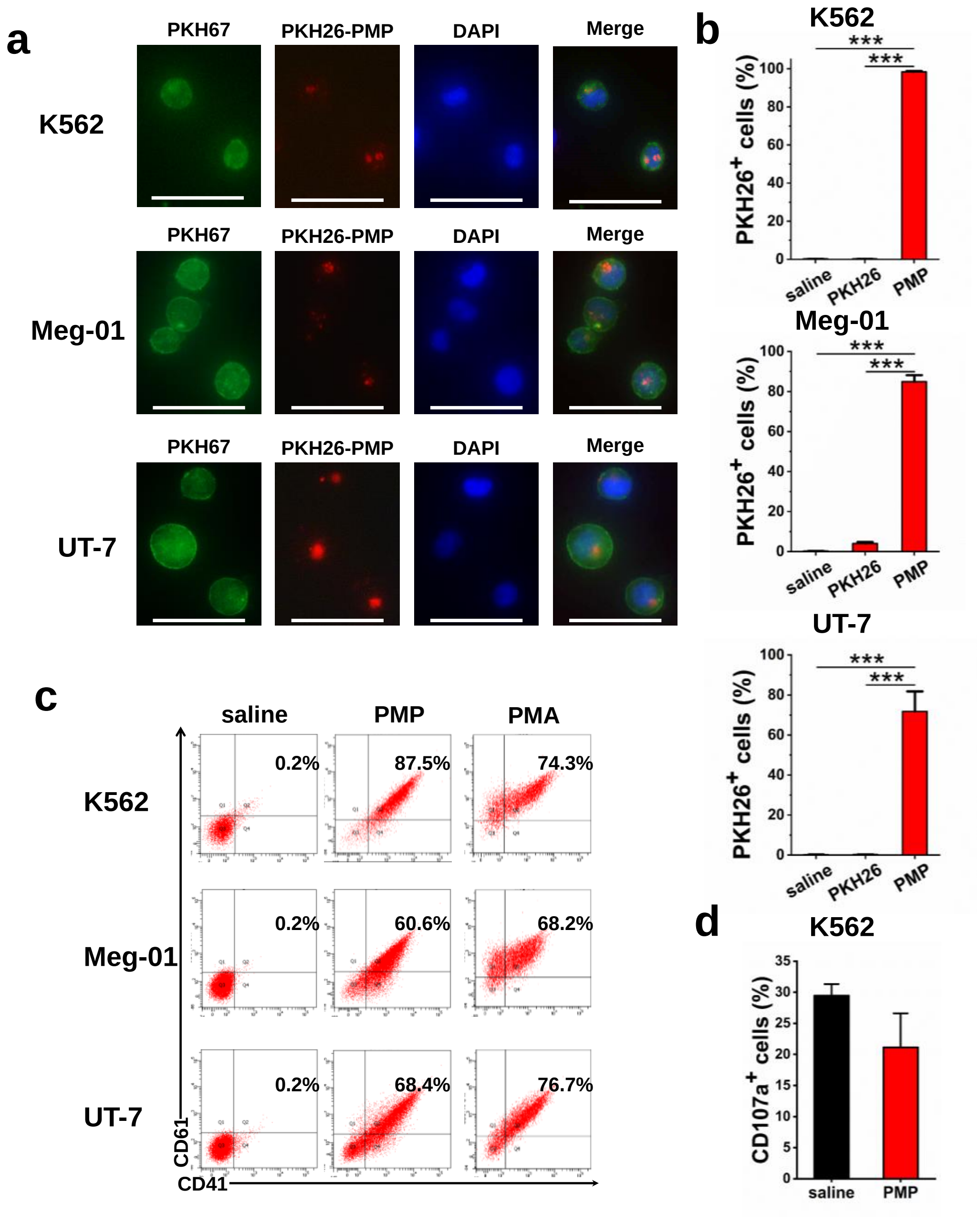

Supplementary Figure 3 PMPs were internalized by K562, Meg-01 and UT-7 cells within 1 day and induced megakaryocytic differentiation in these cells.

(a) An epifluorescence microscopy image shows that 24 hours after PMP addition, the K562, Meg-01, UT-7 cells had internalized fluorescent PMP stained with PKH-26 and exhibited diffuse fluorescence. (Scale bar: 50 nm). (b) Flow cytometry analysis showed that the majority of cells had internalized the isolated PMPs. Cells treated with saline or aggregated fluorescent dye was negative control. One-way ANOVA with Bonferroni's multiple comparison tests were used for statistical analysis, $p<0.0001$. (c) Representative flow cytometry plots of three megakaryocytic lineage cells after PMPs or PMA treatment. (d) Flow cytometry analysis of CD107a expression on K562 cells 24 hours after PMP treatment. Unpaired-samples t-tests were used for statistical analysis. All data are expressed as the mean ± S.D. from three independent experiments, P-value. Source data are provided as a Source Data file ***$P<0.001$.

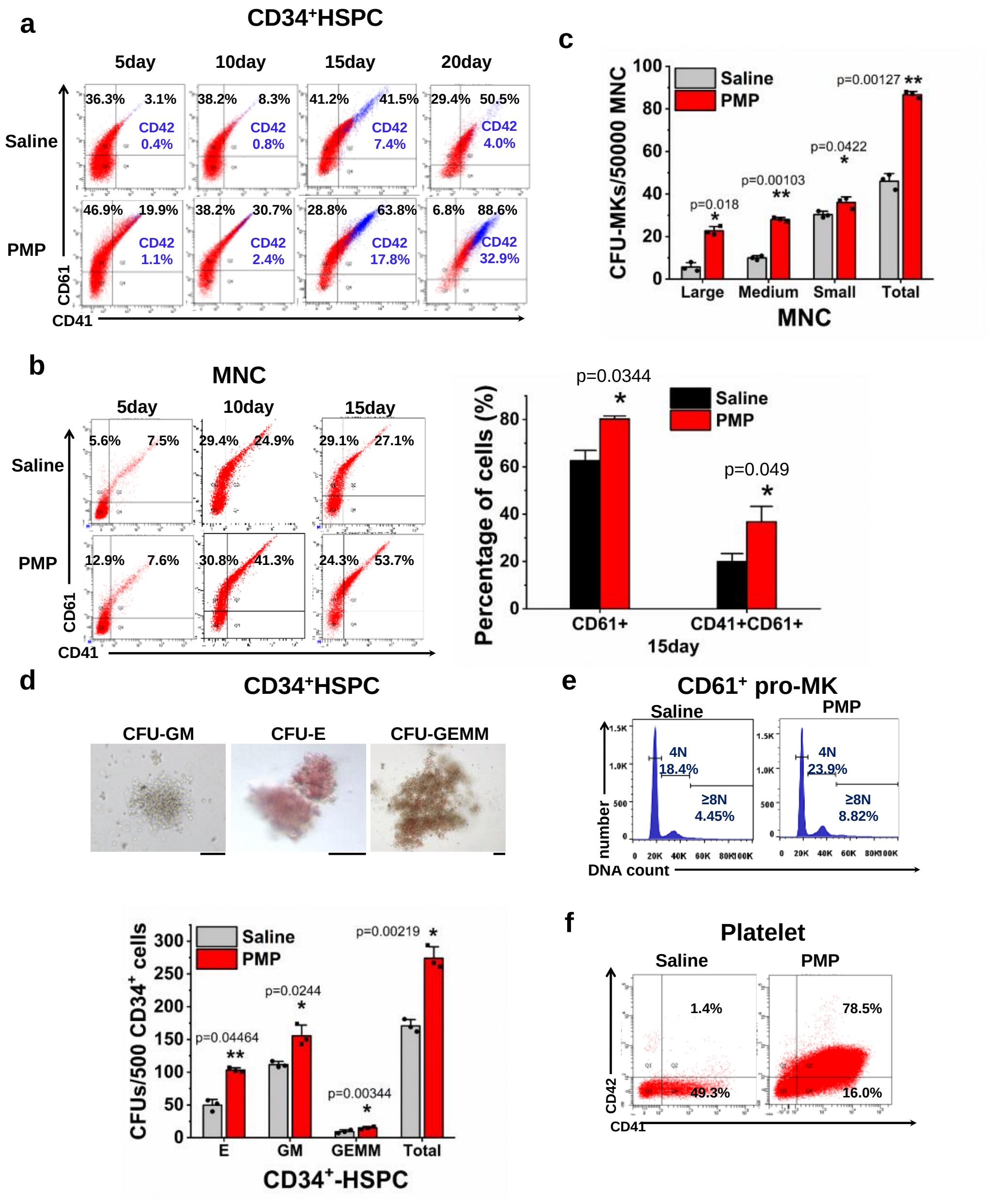

Supplementary Figure 4 Flow cytometric analysis and colony-forming assays demonstrate effects of PMP application on CD34$^+$-HSPC and MNC differentiation.

(a) Representative flow cytometry plots of CD34$^+$-HSPCs megakaryocytic differentiation at indicated time point. Blue dots represent CD41+/CD61+/CD42+ cells. PMP supplement improved megakaryocytic surface marker expression. (b) The effect of PMP internalization on the development of MKs (CD61$^+$ and CD41$^+$CD61$^+$) from MNCs. Representative flow cytometry plots are shown on the left (n=4 donors, mean ± S.E.M). (c) Calculation of CFU-MKs from MNCs in the presence or absence of PMP treatment with megacul colony-forming assays (n=3 donors). (d) The typical morphology of CFU-E, CFU-GM, CFU-GEMM and the quantification of CFUs from CD34$^+$-HSPCs in the presence or absence of initiated PMP treatment with methylcellulose-based colony-forming assays (n=3 donors, scale bar=100 nm) (e) A representative experiment of DNA ploidy analysis by flow cytometry with CD61 positive promegakaryocytes. (f) Representative flow cytometry plots of platelets released in culture media from CD61 positive promegakaryocytes. Cells were obtained from unique cord blood

donors, and each sample was tested with three independent experiments for data presentation. All data are expressed as the mean ± S.D. Two tailed paired-samples t-tests were used for statistical analysis. Source data are provided as a Source Data file * p<0.05, ** p<0.01 and *** p<0.001.

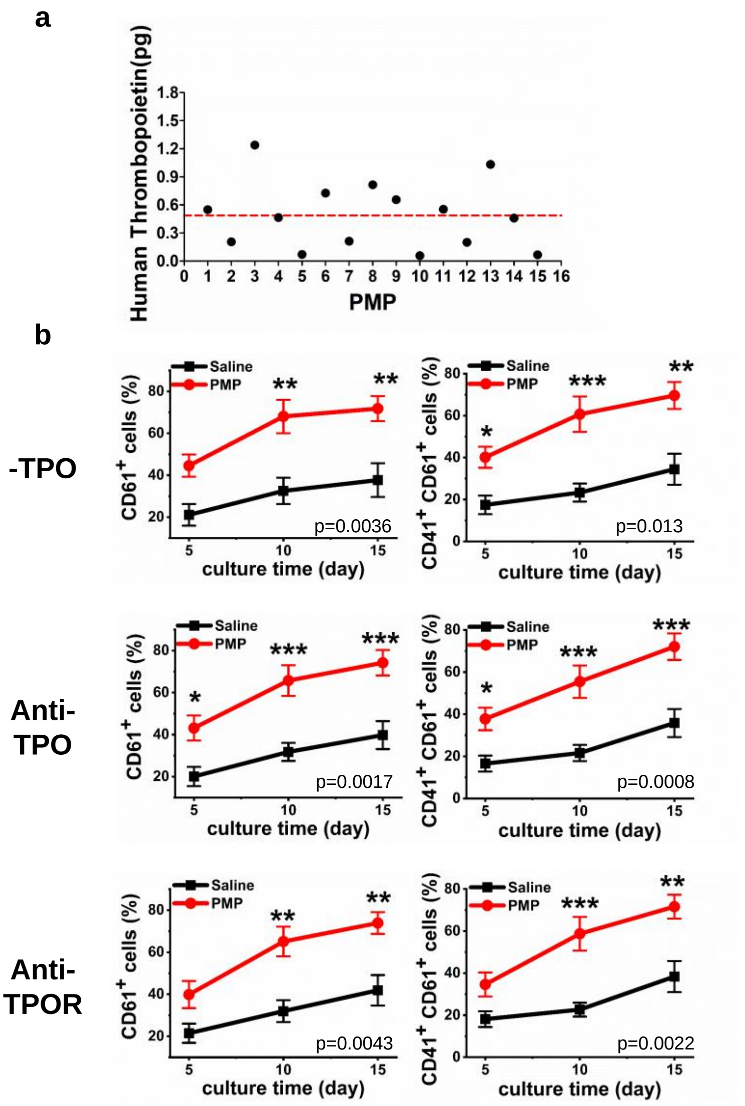

Supplementary Figure 5 Depletion of TPO does not affect the promoting effect of PMPs on MK differentiation.

(a) Concentration of TPO in fresh isolated PMPs determined by ELISA, n=15 mice from three independent experiments. Red line represents mean value (0.4874pg/1×10^5 PMPs). (b) The proportion of CD41+ and CD41+/CD61+ cells of CB-MNCs during megakaryopoiesis measured by FCM. The cells were treated with or without PMPs on cultured day 0 in the absence of TPO (-TPO) or in the presence of TPO neutralizing antibodies (Anti-TPO) or in the presence of TPO receptor’s neutralizing antibodies (Anti-TPOR) (n=6, two-way repeated measures ANOVA with Bonferroni's multiple comparison tests). All data are expressed as the mean ± S.E.M, P-value. Source data are provided as a Source Data file *P<0.05, ** P<0.01, ***P<0.001.

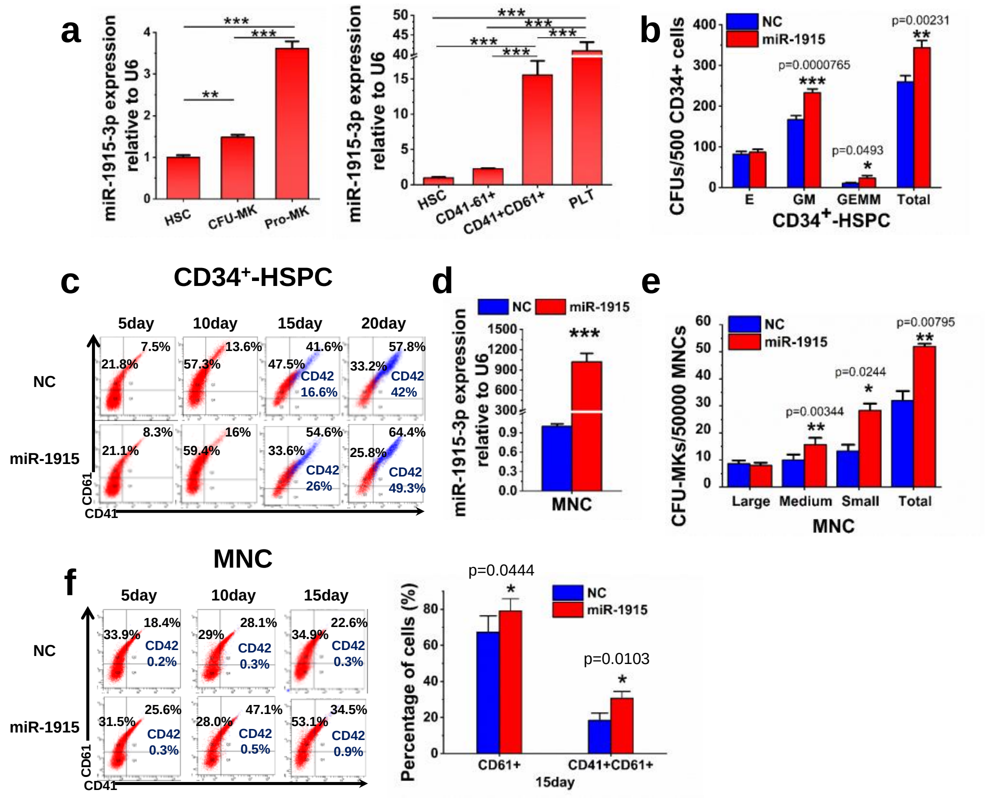

Supplementary Figure 6 miR-1915-3p is a positive regulator of megakaryopoiesis, similar to PMPs which promote HSPCs megakaryocytic differentiation even by day 0 supplement.

(a) Endogenous miR-1915-3p expression increases during MK differentiation. Changes in miR-1915-3p level were evaluated by qPCR. Comparative miRNA real-time PCR was performed in triplicate, and the expression levels were normalized to U6 miRNA. The error bars represent S.D. (n=3 donors, one-way ANOVA with Bonferroni's multiple comparison test, p<0.0001). (b) Quantification of CFUs from miR-1915-3p and NC-modified $CD34^+$-HSPCs with methylcellulose-based colony-forming assays (n=3 donors). (c) A representative experiment of MK integrin CD41, CD61 and CD42 expression during megakaryopoiesis analyzed by flow cytometry. MK-specific induction starts with primary human $CD34^+$-HSPCs. (d) MNCs are transiently transfected with the miR-1915-3p and NC mimics for 24 hours. Relative miR-1915-3p expression is analyzed by qPCR. miR-1915-3p expression is normalized to U6 (n=3 donors, unpaired-samples t-tests,p=0.000136). (e) Calculation of CFU-MKs from miR-1915 and NC modified MNCs with megacul colony-forming assays. (n=3 donors) (f) Overexpression of miR-1915-3p in MNCs induces the expression of MK integrins CD41 and CD61 on day 15 (mean ± S.E.M of 4 donors.). Representative flow cytometry plots at different megakaryopoiesis time points are shown on the left. Cells were obtained from different donors, and each sample was tested with three independent experiments. All data are expressed as the mean ± S.D of three or four unique cord blood donors, Two tailed paired-samples t-tests is used for statistical analysis, unless otherwise specified. P-value. Source data are provided as a Source Data file *P<0.05, ** P<0.01, ***P<0.001.

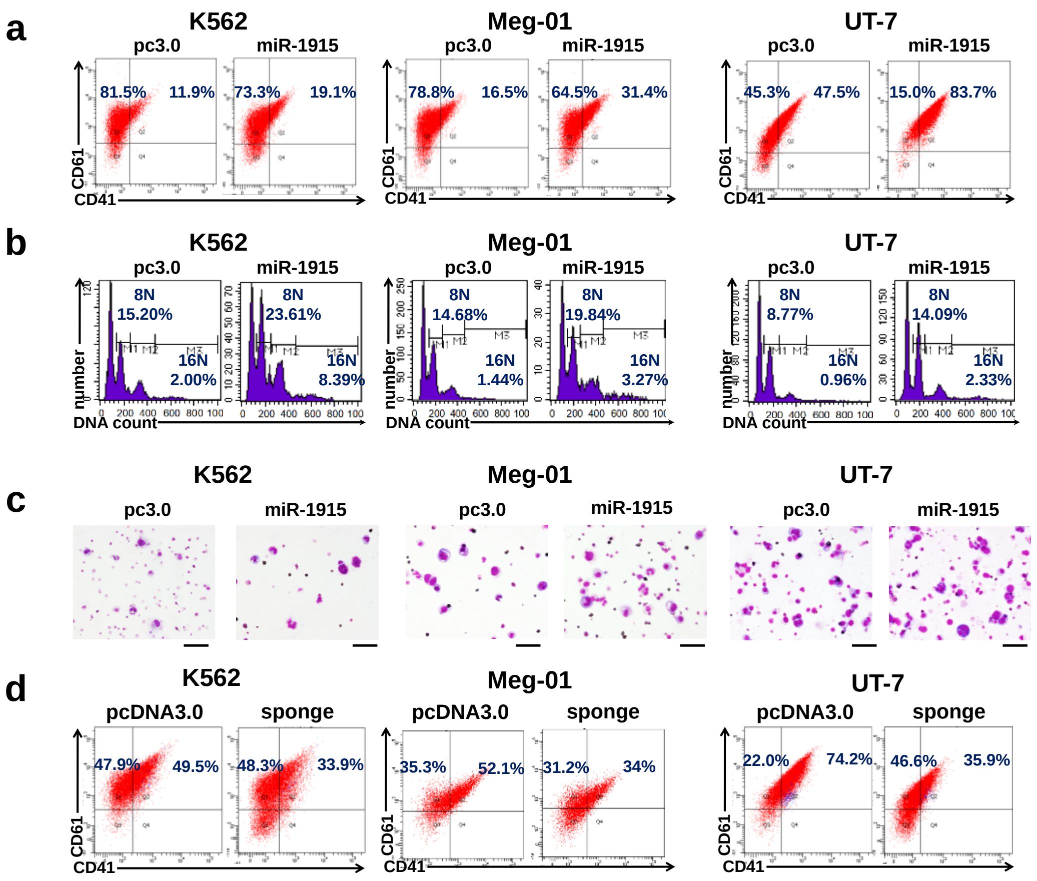

Supplementary Figure 7 Representative flow cytometric plots and microscopy images demonstrate effects of miR-1915-3p modification on megakaryocytic surface marker expression and megakaryocyte morphology.

(a) Representative flow cytometry plots of three megakaryocytic strains stably transfected with pcDNA3.0-neomycin (pc3.0) or pcDNA3.0-pri-miR-1915-3p-neomycin (pc-1915) and treated with a suboptimal amount of PMA (1 nM) for 3 days. (b) A representative experiment of DNA ploidy analysis by flow cytometry of three leukemia lines stably transfected with pc3.0 or pc-1915 and treated with PMA. (c) Morphology change in stable miR-1915-3p-overexpressing leukemia lines in the presence of PMA (10 nM). Cytospin-prepared MKs were stained with Wright–Giemsa solutions (Scale bars: 50 μm). (d) Representative flow cytometry plots of K562, UT-7 and Meg-01 cells stably transfected with pcDNA3.0 and pcDNA3.0-pri-miR-1915-3p-sponge-neomycin (sponge) followed by treatment with 1 nM PMA for 3 days.

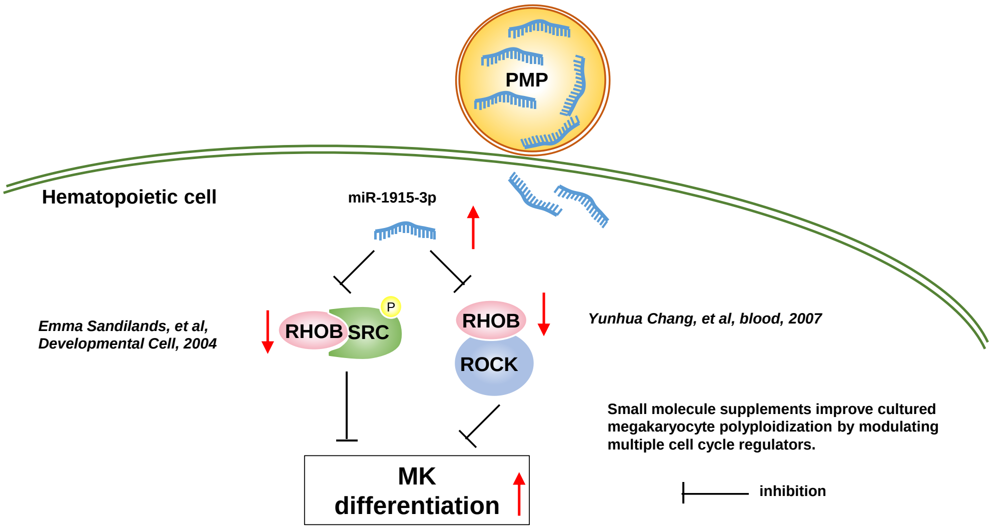

Supplementary Figure 8 Schematic model depicts the internalization of PMPs and the regulatory role of PMPs in megakaryocytic differentiation. The access and internalization of PMPs to their target hematopoietic cells transfers their contented miRNAs. miR-1915-3p is one of the PMP highly enriched miRNAs, which can be transferred and functions in the target cells. The upregulation of miR-1915-3p inhibits *RHOB* expression through specific binding to its mRNA 3'UTR. It has been reported that the interaction and activation of *SRC* or *ROCK* with *RHOB* hinders megakaryocyte differentiation. As a consequence, the upregulation of miR-1915-3p followed by the downregulation of *RHOB* improves MK differentiation.

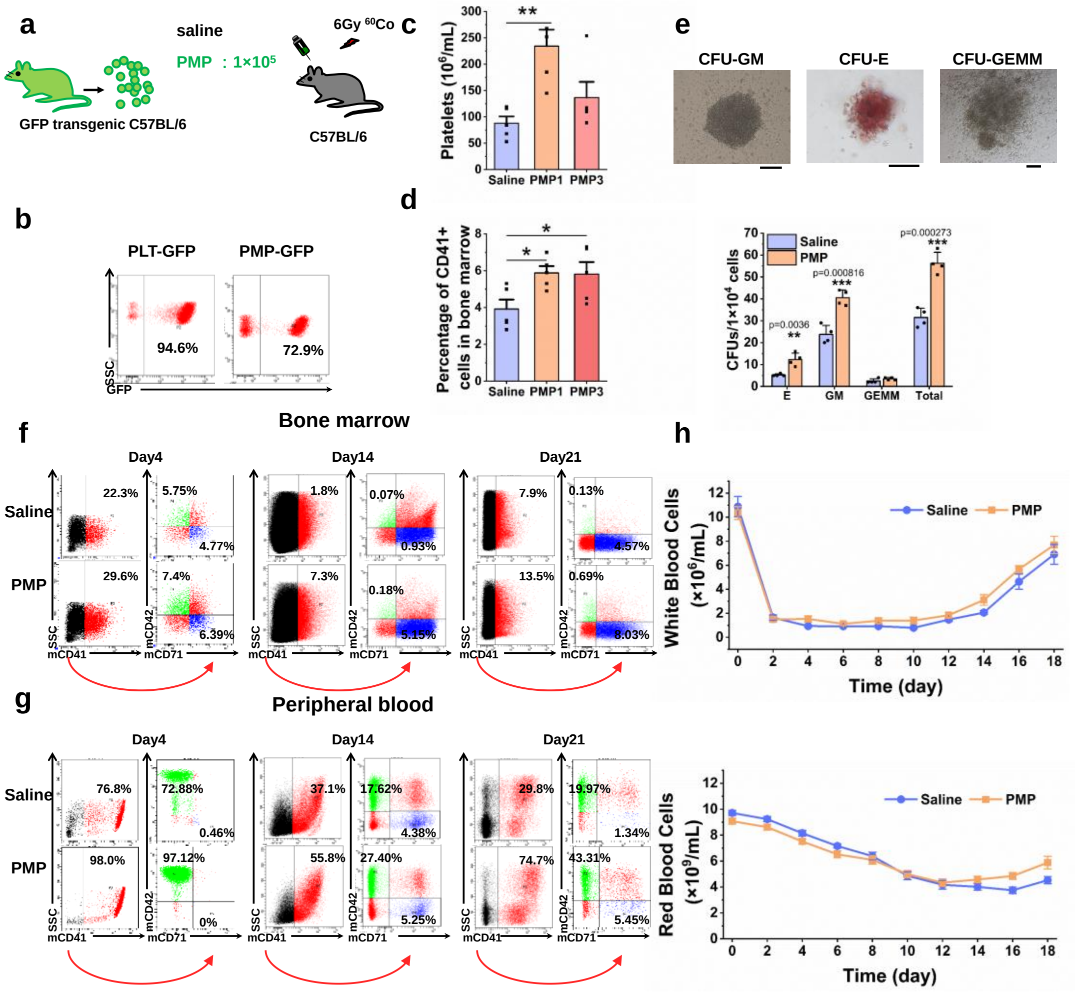

Supplementary Figure 9 Flow cytometric analysis of mouse nucleated cells demonstrates effect of PMP injection in irradiated mice on megakaryocytic surface marker expression.

(a) Experimental scheme of PMP injection to irradiated mice. Semi-lethal dose irradiated mice were transplanted with 1×10^5 GFP-PMPs or an equivalent volume saline right after irradiation. (b) Representative flow cytometry plots of platelets and PMPs from one GFP transgenic C57BL/6 donor mouse. (c, d) After irradiation and injection of 1×10^5 or 3×10^5 WT PMPs or saline, platelet count in peripheral blood on day 14 (p=0.0057) (c) and percentage of whole megakaryocytic cells in bone marrow on day 21(p=0.034) (d).(n=5 mice, One-way ANOVA with Dunnett's multiple comparison test) (e) Mouse BM nucleated cells were analyzed for colony-forming ability and represented in a histogram plot, 3 days after irradiation and injection of 1×10^5 WT PMPs or saline. The typical morphology of mouse CFU-E, CFU-GM, CFU-GEMM are shown on top (n=4 mice, scale bar=100 nm). (f, g) Representative flow cytometry plots of nucleated cells from bone marrow (f) or peripheral blood (g) are shown. Similar to Figure 1c, the proportion of CD41+/CD71+ and CD41+/CD42+ cells were measured from whole CD41 positive cells. (h) Counts of white blood cells and red blood cells in peripheral blood (n=12 mice, two-way repeated measures ANOVA with Bonferroni's multiple comparison tests). Each time point in each animal cohort represents a mean ± S.E.M. Two tailed unpaired t-tests were used at each time point, Source data are provided as a Source Data file *P<0.05, ** P<0.01, ***P<0.001.

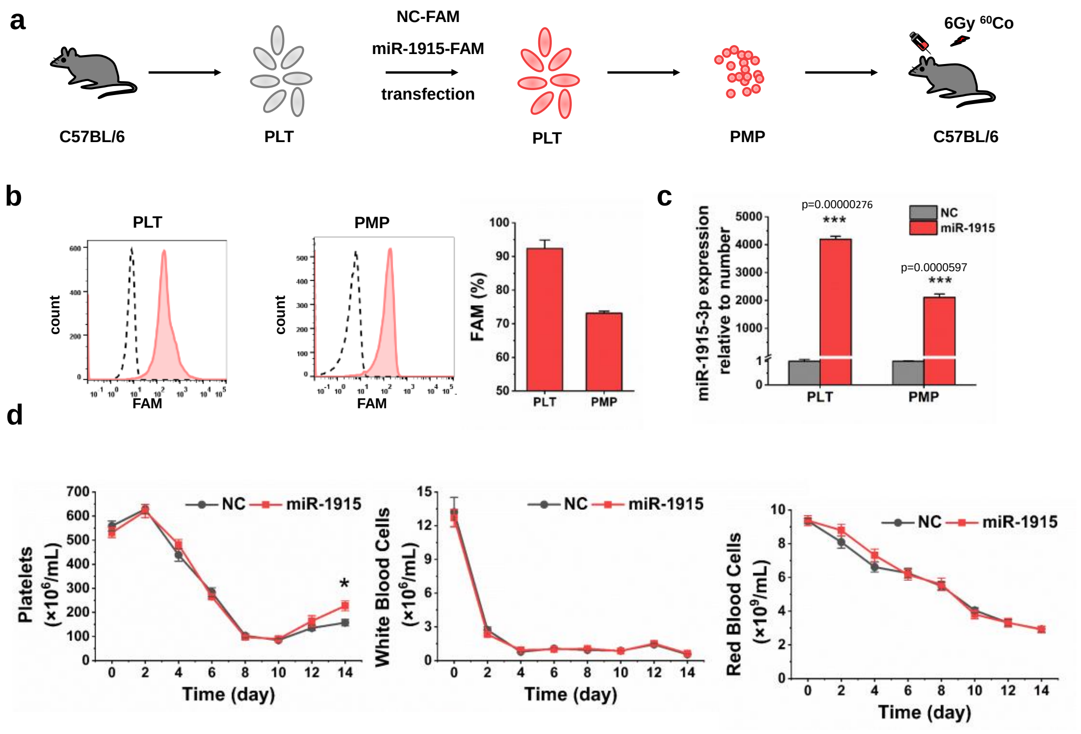

Supplementary Figure 10 Overexpression of miR-1915-3p promotes megakaryocytic differentiation and platelet production in irradiated mice.

(a) Experimental scheme of isolation and injection of PMPs from miRNA transfected platelets. (b) The transfection efficiency of platelets and PMPs confirmed by flow cytometry examination. After transfection, the proportions of FAM-positive events in the platelet and PMP gates were 92.38% and 73.13%, respectively (n=6 mice) (c) After transfection, successful overexpression of mature miR-1915-3p in both platelets and PMPs was confirmed by qPCR analysis. (n=3 mice) (d) Counts of white blood cells, red blood cells and platelets in peripheral blood (n=10 mice, two-way repeated measures ANOVA with Bonferroni's multiple comparison tests). Each time point in each animal cohort represents a mean ± S.E.M. Two-tailed unpaired t-tests were used at each time point. Source data are provided as a Source Data file *P<0.05, ** P<0.01, ***P<0.001.

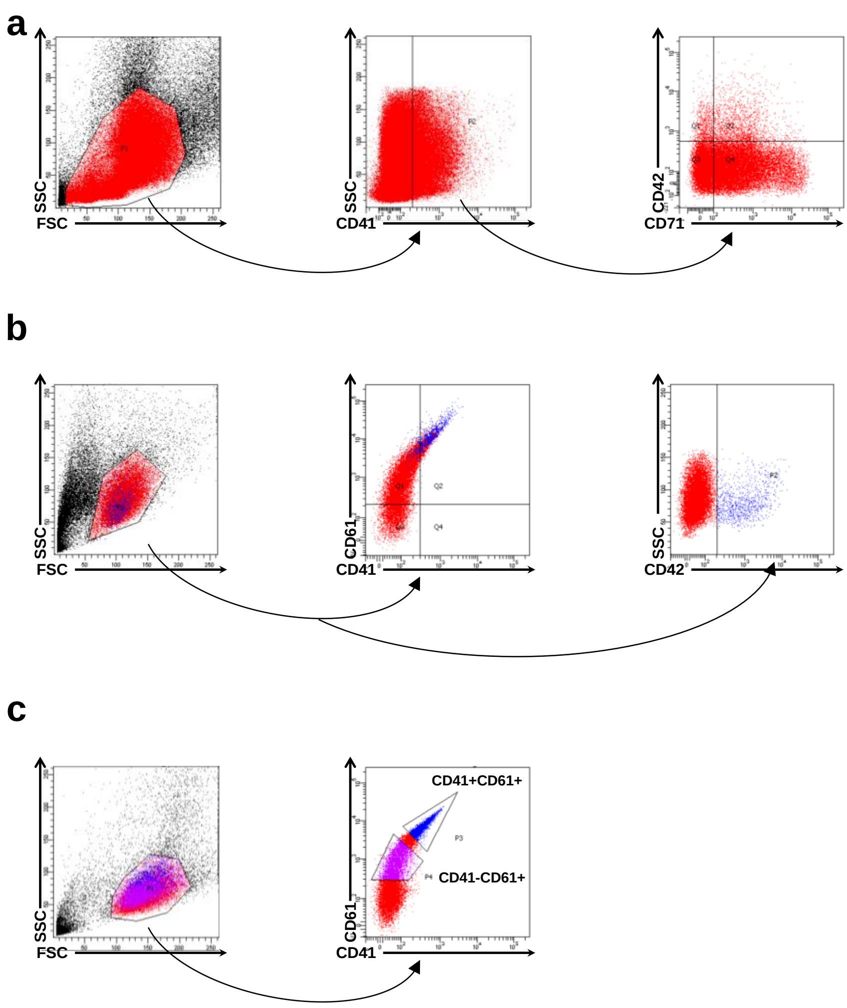

Supplementary Figure 11 Gating or sorting strategies for immunophenotyping of megakaryocytic cell markers.

(a) Gating scheme were used to analyze mouse nucleated cells from bone marrow or peripheral blood after CCl_4 exposure or mouse PMP transplantation in figure 1c, 6c, 6e,6j and supplementary figure 1f, 1g, 9f, 9g. (b) Gating strategy were used to determine the percentage of megakaryocytic cells after MK differentiation in supplementary figure 4a, 6c and 6f. (c) Sorting strategy were used to determine the percentage of $CD41^{+}CD61^{+}$ cells and $CD41^{-}CD61^{+}$ cells after MK differentiation in supplementary figure 6a.

Supplementary Table 1. Primers used for gene amplification

**Gene**	**Primer name**	**Primer Sequence（5’-3’）**	**Annealing temperature (∘C)**
mTpo	mTpo F<br>mTpo R	GTGGCAAGACTAACTCTGTCC<br>GGAGTCACGCAGCAGTTTATTTA	55℃
Htrp	Htrp F<br>Htrp R	TCAGTCAACGGGGGACATAAA<br>GGGGCTGTACTGCTTAACCAG	55℃
RHOB	RHOB F<br>RHOB R	CTGCTGATCGTGTTCAGTAAGG<br>TCAATGTCGGCCACATAGTTC	58℃
GAPDH	GAPDH F<br>GAPDH R	GAGTCAACGGATTTGGTCGT<br>TTGATTTTGGAGGGATCTCG	58℃
DHCR24	DHCR24 F<br>DHCR24 R	GCCGCTCTCGCTTATCTTCG<br>GTCTTGCTACCCTGCTCCTT	58℃
LSS	LSS F<br>LSS R	GCACTGGACGGGTGATTATGG<br>TCTCTTCTCTGTATCCGGCTG	58℃
PML	PML F<br>PML R	GGATGAAGTGCTACGCCTCG<br>TCCCCTGGGTGATGCAAGA	58℃
TRIM7	TRIM7 F<br>TRIM7 R	GCTCGGGGTTGAGATCACC<br>CCAGGCACATTGCTACACCT	58℃
C6orf223	C6orf223 F<br>C6orf223 R	CCTTCAGCAACGGAGTGGG<br>ACTGCTTTCCTATGTCTGGGG	58℃
PTPRF	PTPRF F<br>PTPRF R	GCTTCGAGGTCATTGAGTTTGA<br>CCCATGTCGATGGAAGGGAA	58℃
SCD	SCD F<br>SCD R	TCTAGCTCCTATACCACCACCA<br>TCGTCTCCAACTTATCTCCTCC	58℃
BTN2A2	BTN2A2 F<br>BTN2A2 R	GGGCCAGCTAATCCCATCC<br>GGTGATTCTTCCCCGGTACTC	58℃

F: forward primer, R: reverse primer

Supplementary Table 2 Antibodies used in our study. Detailed information of antibodies used in our study.

Antibody	Supplier name	Catalog number	Clone Name	Lot Number	Application	Dilution
FITC-anti-mouse-CD41	eBioscience	11-0411	MWReg30	2111527	FCM	5 μL (0.5 μg)/test
APC-anti-mouse-CD42d	eBioscience	17-0421	1C2	E10884-1636	FCM	5 μL (0.5 μg)/test
PE-anti-mouse-CD71	eBioscience	12-0711	R17217	E01340-1632	FCM	5 μL (0.5 μg)/test
FITC-anti-mouse-CD34	eBioscience	11-0341	RAM34	E00264-1632	FCM	5 μL (0.5 μg)/test
PE-anti-human-CD34	eBioscience	12-0349	4H11	4299670	FCM	5 μL (0.5 μg)/test
APC-anti-human-CD61	eBioscience	17-0619	VI-PL2	4283816	FCM	5 μL (0.5 μg)/test
FITC- anti-human-CD41a	eBioscience	11-0419	HIP8	E10848-1632	FCM	5 μL (0.5 μg)/test
PE- anti-human-CD41a	eBioscience	12-0419	HIP8	E14074-104	FCM	5 μL (0.5 μg)/test
PE- anti-human-CD42b	eBioscience	12-0429	HIP1	2056924	FCM	5 μL (0.5 μg)/test
anti-mouse-CD41 monoclonal antibody	Abcam	ab33661	MWReg30	GP213177-3	IHC	1:50
β-Actin mouse monoclonal antibody	Cell Signaling Technology	12262	8H10D10	2	WB	1:1000
GAPDH monoclonal antibody	Cell Signaling Technology	8884	D16H11	3	WB	1:1000
RHOB polyclonal antibody	Abclonal	A2819		1151540301	WB	1:1000
CD63 mouse monoclonal antibody	Santa Cruz biotechnology	sc-365604	E-12	F2110	WB	1:200
Human thrombopoietin antibody	R&D Systems	MAB288	#34817	AYH0315021	Neutralization	1 μg per mL
Human thrombopoietin receptor antibody	R&D Systems	AF1016		GSS0315121	Neutralization	1 μg per mL
